# A National Surveillance of the Antibiotic Susceptibility of *Acinetobacter baumannii* in Saudi Arabia

**DOI:** 10.3390/antibiotics14020209

**Published:** 2025-02-19

**Authors:** Abrar K. Thabit, Feras S. Alharbi, Anas F. Jawah, Ammar M. Alghamdi, Musaab Y. Miaji, Fatimah Alturki, Nehal Hosin, Mohammed Bazuqamah, Masaad Saeed Almutairi, Hamad Alhamed, Alaa Elhendawy, Dalya Atallah, Abdulaziz A. Humadi, Khalid A. Alfifi, Khadija Alfadel, Khalid Eljaaly

**Affiliations:** 1 Department of Pharmacy Practice, Faculty of Pharmacy, King Abdulaziz University, Jeddah 22254, Saudi Arabia; 2 Faculty of Pharmacy, King Abdulaziz University, Jeddah 22254, Saudi Arabia; 3 Microbiology Department, King Fahad Hospital of the University, Khobar 34445, Saudi Arabia; 4 Department of Microbiology, College of Medicine, Imam Abdulrahman bin Faisal University, Dammam 34212, Saudi Arabia; 5 Microbiology Department, King Khaled Hospital, Najran 66262, Saudi Arabia; 6 Department of Pharmacy Practice, College of Pharmacy, Qassim University, Qassim 51452, Saudi Arabia; 7 Laboratory and Blood Bank Department, King Fahad Specialist Hospital, Qassim 52366, Saudi Arabia; 8 Microbiology Department, King Fahad Hospital, Albaha 65732, Saudi Arabia; 9 Department of Clinical Microbiology, King Abdulaziz University Hospital, Jeddah 22254, Saudi Arabia; 10 Laboratory and Blood Bank Department, King Fahad Specialist Hospital, Tabuk 47717, Saudi Arabia; 11 Microbiology Department, King Fahad Specialist Hospital, Tabuk 47717, Saudi Arabia; 12 Microbiology Department, Maternity and Children Hospital, King Salman Medical City, Madinah 42319, Saudi Arabia

**Keywords:** *Acinetobacter baumannii*, antibiotics, antimicrobial resistance, broth microdilution, surveillance, Saudi Arabia

## Abstract

Most surveillance studies in Saudi Arabia have been single-centered or did not use the gold standard broth microdilution (BMD) antimicrobial susceptibility test. This is the first study from Saudi Arabia to evaluate the resistance profiles of *Acinetobacter baumannii* by using BMD on a national level. Between November 2022 and April 2023, isolates from several infection sites were collected from seven hospitals in seven regions of Saudi Arabia. On testing days, BMD was done following Clinical Laboratory Standards Institute standards. Antibiotic susceptibility percentages and MIC_50_ and MIC_90_ were calculated. One hundred *A. baumannii* isolates were included. The highest susceptibility was to tigecycline (39%) and aminoglycosides (22–25%). The MIC_90_ of all antibiotics were higher than the resistance breakpoint. All isolates (100%) were multidrug-resistant, of which 52% were classified as extensive-drug-resistant, and 42% were identified as pandrug-resistant. The isolates collected from the ear, peritoneal fluid, and the cerebrospinal fluid were all XDR, while 2/3 of the urine isolates (10/15; 66.7%), more than 1/2 of the skin/soft tissue and respiratory isolates (9/16; 56.3% and 22/43; 51.7%, respectively), and 3/8 (37.5%) of the blood isolates met this definition. Conversely, PDR isolates made up 5/8 of blood isolates (62.5%), 8/15 of body fluid isolates (57.14%), and 19/43 (44.2%) of respiratory isolates. *A. baumannii* showed a surprisingly high resistance to multiple commonly used antibiotics. Infection control policies and antimicrobial stewardship should be implemented by hospitals throughout the country to improve treatment, track resistance trends with local antibiograms, and prevent the development of resistant strains.

## 1. Introduction

*Acinetobacter baumannii*, a non-fermentative Gram-negative coccobacillus from the Moraxellaceae family, has resulted in increasing global attention as a nosocomial pathogen in recent years. Over the previous decade, *A. baumannii* strains, often displaying multidrug resistance, have emerged as a prominent global health challenge as they have been implicated in various infections, primarily of nosocomial origin [1]. The prevalence of *A. baumannii* is particularly pronounced in intensive care units, where controlling numerous outbreaks has proven exceptionally challenging [2]. *A. baumannii* is intrinsically resistant to numerous antibiotics, including ampicillin, ertapenem, aztreonam, and fosfomycin [3].

Bacterial resistance to antimicrobial agents is a growing global concern that significantly impacts both patient clinical outcomes and the economic stability of hospitals and governments [1]. A list of antibiotic-resistant priority pathogens was published by the World Health Organization (WHO) in 2024, classifying them as either critical, high, or medium priority [4]. In this list, carbapenem-resistant *A. baumannii* was categorized under “critical priority”, which indicates that this organism poses a threat to patients due to their association with deadly infections, such as bacteremia and pneumonia. In a recent review article of antimicrobial resistance (AMR) in Saudi Arabia between 2013 and 2023 that included nine studies evaluating antibiotic resistance by *A. baumannii*, the reported high resistance rates have increased over the last decade [5]. In fact, all the reported isolates were multidrug-resistant (MDR). The antibiotic to which the isolates (*n* = 1082) exhibited the highest susceptibility rates was colistin (97.5%), though it should be noted that such susceptibilities were tested using automated susceptibility testing (AST) methods, such as Vitek2, Phoenix, and Microscan, rather than the recommended reference method of broth microdilution (BMD) [6,7,8], except in one study [5,9]. Conversely, the susceptibility to ciprofloxacin was the lowest (1.25%). Furthermore, the review revealed elevated resistance to carbapenems, including to imipenem and meropenem, with average percentages of susceptibility below 20%.

Numerous methods for testing antimicrobial susceptibility have been documented in the literature. However, only BMD stands out as the ‘gold standard’ according to international laboratory standards [6,7,8]. Since no study in the literature from Saudi Arabia has evaluated the susceptibility of *A. baumannii* using the gold standard method of BMD, we aimed to test the susceptibility of *A. baumannii* on a national level against a panel of antibiotics. Our aim was to be a leading project to report AMR rates of *A. baumannii* on a large scale in Saudi Arabia by including isolates from various hospitals across the country. Another objective of the study was to ultimately contribute a scientifically grounded foundation to advocate for the formulation and implementation of antimicrobial stewardship programs within Saudi hospitals, as well as foster the adoption of infection control and prevention strategies to further enhance healthcare practices in the region.

## 2. Results

### 2.1. Isolates Distribution by Participating Hospitals

A total of 100 *A. baumannii* isolates were collected, mostly from Dammam (23%), followed by Qassim (20%) and Tabuk (20%), as shown in Table 1.

### 2.2. Sources of Isolates

The sources of the isolates are presented in Table 2, where most isolates came from a respiratory source (43%), skin and soft tissue (16%), urine (15%), or body fluids (14%). All the isolates (100%) were MDR, whereas 52% and 42% of isolates were XDR and PDR, respectively.

### 2.3. Susceptibility Results

Table 3 shows the susceptibility percentages and MIC data of the isolates. Susceptibility was the highest to tigecycline (39%) with MIC_50_ and MIC_90_ of 4 and >8 μg/mL, respectively. Aminoglycosides ranked second to tigecycline in terms of activity, with the highest percentage susceptibility being observed with tobramycin (25%), followed by gentamicin (23%) and amikacin (22%). In contrast, susceptibility to the remaining tested antibiotics was extremely low, reflecting a high resistance rate to β-lactams (including ampicillin/sulbactam and carbapenems), fluoroquinolones, and polymyxins.

**Figure 1:**
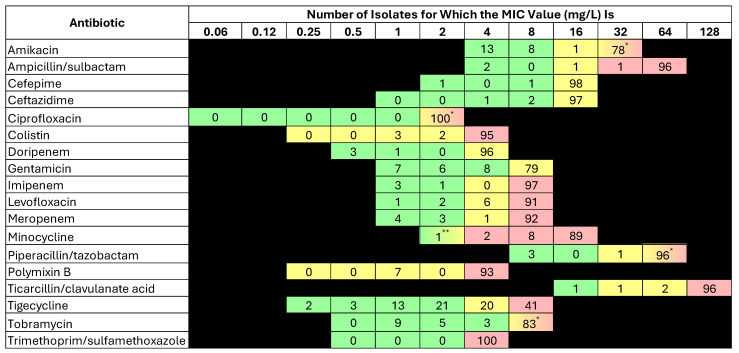
Number of isolates reported for each MIC value. The color shades reflect the susceptibility breakpoints according to CLSI standards [7], except tigecycline, which’s breakpoints is based on US FDA breakpoint. The green shade indicates susceptibility, the yellow shade indicates intermediate susceptibility, and the red shade indicates resistance. * The intermediate susceptibility breakpoint begins at this value, followed by resistance at the next dilution. ** The susceptibility breakpoint begins at this value, followed by intermediate susceptibility at the next dilution.

Figure 1 shows the number of isolates reported for each MIC value, with color shades reflecting CLSI breakpoints for each antibiotic.

### 2.4. Distribution of Drug-Resistant Isolates

**Figure 2:**
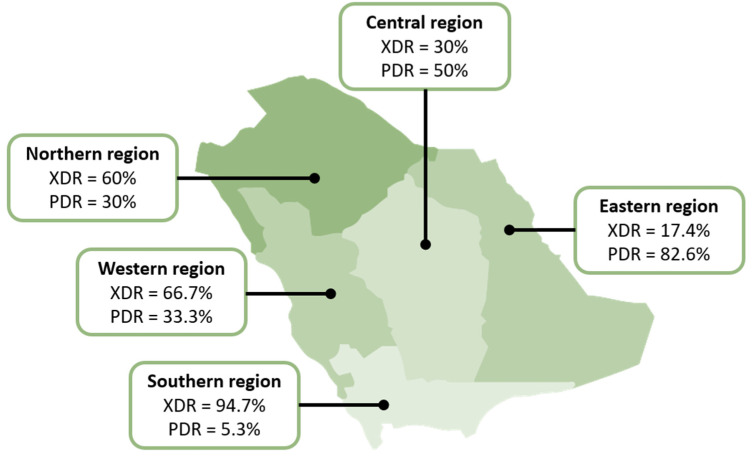
Distribution of XDR and PDR *Acinetobacter baumannii* isolates in Saudi Arabia.

Of the 100 isolates collected, all met the definition of MDR, 52% were XDR, and 42% were PDR. The distribution of these resistance patterns based on the region is shown in Figure 2, where a significant difference was observed between the regions in the distribution of XDR and PDR isolates (*p* < 0.0001 for both resistance patterns). The isolates collected from the ear, peritoneal fluid, and the cerebrospinal fluid were all XDR, while 2/3 of the urine isolates (10/15; 66.7%), more than 1/2 of the skin/soft tissue and respiratory isolates (9/16; 56.3% and 22/43; 51.7%, respectively), and 3/8 (37.5%) of the blood isolates met this definition. PDR isolates made up 5 of the 8 blood isolates (62.5%) and 8 of the 15 body fluid isolates (57.14%). Of the 43 respiratory isolates, 19 (44.2%) were PDR. No statistical difference was observed in the distribution of XDR isolates (*p* = 0.512) and PDR isolates (*p* = 0.425) between infection sites (Table 2). The exact MICs and susceptibility results are provided in the Supplementary Table (Supplementary Table S1: MIC results of all tested antibiotics against *A. baumannii*).

## 3. Materials and Methods

### 3.1. Study Period and Collection Process

Seven large hospitals from seven administrative regions of Saudi Arabia participated in this study, where unique *A. baumannii* clinical isolates from various infection sites were collected between November 2022 and April 2023. All isolates were transported on blood agar plates (BAPs) (Saudi Prepared Media Laboratory, Co., Ltd., Riyadh, Saudi Arabia) to a central microbiology laboratory (at the Faculty of Pharmacy, King Abdulaziz University, Jeddah). Ethical approval was obtained from the Saudi Ministry of Health (reference no. 607/44/5113) and the Biomedical Research Ethics Committee of the Faculty of Medicine of King Abdulaziz University (ref No. 559–22).

### 3.2. Bacterial Isolates

Consecutive clinical isolates of *A. baumannii* labeled with the infection site were prospectively collected by the participating hospitals. Isolate identification was done at each hospital using automated antimicrobial susceptibility testing methods, such as BD Phoenix 100 (Becton, Dickinson and Company, Franklin Lakes, NJ, USA) or VITEK 2 (bioMérieux, Craponne, France). Upon reception, the isolates were subcultured on BAPs, then stored at −80 °C in glycerol until the testing day. Isolates that exhibited poor growth patterns were subcultured again from the newly grown colonies to ensure the storage of sufficient bacterial counts. Before antibiotic susceptibility testing, the isolates to be tested were thawed and then subcultured on BAPs and incubated for 24 h at 36 °C.

### 3.3. Antimicrobial Susceptibility Testing

Sensititre GNX3F plates (Thermo Fisher Scientific, Waltham, MA, USA) were used to conduct BMD according to the Clinical Laboratory Standards Institute (CLSI) standard (M100–33) [3]. The list of antibiotics included in these plates is shown in Table 3. These plates comprise a panel of various antibiotics against Gram-negative bacteria. To ensure the accuracy of the plates, the first microtiter plate of the batch was assessed using ATC27853 (*Pseudomonas aeruginosa* quality control isolate). On testing days, 50 μL of prepared Mueller–Hinton Broth (Biolab, Inc., Budapest, Hungary) were instilled in the microtiter plates. In the meantime, a bacterial inoculum was prepared with a density of 0.5 McFarland (equivalent to 1.5 × 10^8^ CFU/mL). The density of the prepared inocula was confirmed using a daily calibrated densitometer (Grant Instruments, Ltd., Royston, UK). A volume of 10 μL of the inoculum was then instilled in the microtiter plates, followed by incubation for 16–24 h at 36 °C prior to reading.

### 3.4. Definitions

An organism is labeled as MDR if it exhibits resistance to three or more antibiotics from different classes, whereas isolates that are resistant to at least one agent in all but 2 or fewer antimicrobial classes are labeled extensive drug-resistant (XDR) [10]. An isolate that is resistant to all antibiotics from all antibiotic classes is known as pandrug-resistant (PDR) [10]. Of note, these terms apply to acquired resistance against antibiotics, to which wild-type *A. baumannii* is susceptible (i.e., not intrinsically resistant).

### 3.5. Data Analysis

The percentages of susceptible organisms (% susceptibility) to each antibiotic, minimum inhibitory concentrations required to inhibit 50% of the isolates (MIC_50_), MIC required to inhibit 90% of the isolates (MIC_90_), and percentages of different drug-resistant patterns were calculated from the data that were logged in Microsoft Excel (Microsoft, Corp., Seattle, WA, USA). The susceptibility of all antibiotics, except tigecycline, was determined based on CLSI breakpoints (document M100–33) [3]. For tigecycline, the susceptibility breakpoints approved by the US Food and Drug Administration for Enterobacterales that are listed in the drug’s package insert were used for MIC interpretation [11]. The chi-square test was done using SPSS version 24.0 (IBM Corp., Armonk, NY, USA) to compare the distribution of XDR and PDR isolates among regions and infection sites. Since all the isolates were MDR, the test could not be carried out on them.

## 4. Discussion

In this study, 100 isolates of *A. baumannii* were collected from seven hospitals throughout Saudi Arabia as part of the country’s first national multicentric antimicrobial surveillance study. These isolates were obtained from various infection sites, mostly from the respiratory tract, urinary tract, skin/soft tissue, and body fluids. A recent review that evaluated the epidemiology of *A. baumannii* in Saudi Arabia noticed that this pathogen is largely responsible for respiratory tract infections, mostly in intensive care units (ICUs) [12]. This is probably because *A. baumannii* is a major contaminant of respiratory support equipment and suction devices commonly used in ICUs [13].

While infection types were not collected, the knowledge of infection sites could provide an insight into the type of infections the patients had due to *A. baumannii*. The most common site was the respiratory tract, which implies that pneumonia was prevalent among the patients from whom the isolates were collected. This aligns with the previous knowledge that nosocomial pneumonia is the top infection caused by *A. baumannii*, especially in critically ill patients [14]. Interestingly, only eight isolates were from the blood (a common infection site for *A. baumannii*) compared with 16 from skin and soft tissue and 15 from the urinary tract. Most skin infections due to *A. baumannii* are usually surgical site infections [14,15].

Results from our surveillance study revealed high susceptibility to aminoglycosides compared to carbapenems, which are considered the broadest-spectrum β-lactams. Moreover, tigecycline exhibited the highest in vitro activity, whereas ciprofloxacin and trimethoprim/sulfamethoxazole demonstrated the lowest activity. In our study, all *A. baumannii* isolates (100%) were MDR, whereas 52% and 42% of isolates were XDR and PDR, respectively. A recent review that evaluated AMR among various bacterial pathogens, including *A. baumannii*, over the last decade showed an increasing prevalence of AMR [5]. The review included nine studies that were mostly single-centered and used automated systems for susceptibility testing. In that review, the highest average susceptibility from all the studies was to colistin (97.5%), followed by amikacin (36.3%). It is worth noting that the discrepancy in the susceptibility rates to colistin between our study and these previous studies could be attributed to the difference in the testing method used (automated susceptibility tests vs. BMD), where BMD is the method recommended by the latest international colistin guidelines [16]. Furthermore, the extremely low susceptibility to colistin demonstrated in our study could be attributed to a phenomenon of the attachment of polymyxins to the plastic bottom of the microtiter plates used in BMD. Previous studies on BMD observed that polymyxins, including colistin and polymyxin B, have an affinity for plastic—the material from which BMD microtiter plates are made [17,18]. This interaction with plastic can affect MIC measurements. To mitigate this effect, Sutherland et al. recommended using polysorbate 80 by adding it to the microtiter plates wells when assessing colistin MIC using BMD. Polysorbate 80 serves as a surfactant, preventing colistin from adhering to the plastic plate bottom. Our study reported a low susceptibility rate to colistin, which may be partly attributed to this phenomenon. However, since our study used pre-filled plates with lyophilized antibiotics, adding polysorbate 80 was not feasible.

A large study from a hospital in Jeddah (in the western region of Saudi Arabia) between September 2013 and May 2014 included 527 *A. baumannii* isolates, of which 244 were respiratory, 93 were urinary, and 190 were from miscellaneous infection sites [19]. In that study, 35.7% of the respiratory isolates and 34.4% of the urinary isolates were susceptible to all tested antimicrobials, while 64.3% and 65.6% were resistant to one or more antimicrobials from these sites, respectively. Of the isolates collected from miscellaneous sites, 28.4% were susceptible to all tested antimicrobials, while 71.6% were resistant to one or more antimicrobials. In another study from the same region by Shah et al. between 2015 and 2016, 58.5% of the 135 *A. baumannii* isolates were MDR, but none were PDR, which is inconsistent with our findings [9]. Similar to our study, the highest susceptibility was to tigecycline, though it was much higher than the value reported in our study (96% vs. 39%). While all isolates were susceptible to colistin in that study, our study showed a very low susceptibility rate (5%) [6]. Nonetheless, it should be noted that these results were generated about a decade ago. Hence, the low susceptibilities shown in our study may indicate the rapid increase in AMR rates. In addition, newer studies from various regions of Saudi Arabia showed higher resistance rates for *A. baumannii* [20,21,22,23]. For instance, a study from the Eastern region (2017–2018) showed that all the collected 103 isolates were MDR exhibiting a substantial resistance to carbapenems (only 1.9% were susceptible to imipenem and 42.7% were susceptible meropenem) and aminoglycosides, with a susceptibility rate reaching around 52% [20]. The highest susceptibility was to tigecycline (95.1%). Another recent study in 2020 from Hail (in the northern part of Saudi Arabia) showed that all the included 82 *A. baumannii* isolates were XDR, with the highest susceptibility being reported to amikacin (61.3%), followed by tigecycline (12.8%), whereas the susceptibility to colistin was only 5%, which matches the rate reported in our study [21]. Using disk diffusion methods, a study from Riyadh (in the central region of Saudi Arabia) showed that all the 54 *A. baumannii* isolates that were collected between 2013–2014 were MDR, though all were susceptible to colistin [24].

The β-lactamase inhibitor sulbactam was used for decades against *A. baumannii* when it was only available as a combination with ampicillin. However, due to the fixed concentration of sulbactam in this combination and to limit the unnecessary exposure to ampicillin, two new combinations of β-lactamase inhibitors involving sulbactam have been developed: sulbactam/durlobactam and sulbactam/avibactam. Sulbactam/durlobactam received US FDA approval in May 2023 for hospital-acquired and ventilator-associated pneumonia caused by *A. baumannii* [25]. In an in vitro study against 5032 *A. baumannii* isolates from 33 hospitals across the globe, 98.3% of the isolates exhibited susceptibility to this combination (based on a presumed susceptibility breakpoint of 4 μg/mL), including MDR and XDR isolates with an MIC_50_ and MIC_90_ of 1 and 2 μg/mL, respectively [26]. Similarly, sulbactam/avibactam (as ampicillin/sulbactam plus ceftazidime/vibactam), has shown potent activity against drug-resistant *A. baumannii*, where 124 of 127 (97.6%) XDR isolates had MICs ≤ 4 μg/mL [27]. This study, as well as another study by Pasteran et al., demonstrated this activity mostly against OXA-producers but less against NDM-producers [28]. In the study by Pasteran et al., 14% of 187 isolates were inhibited by sulbactam alone at 4 μg/mL, but this percentage increased to 69% when sulbactam was combined with avibactam (91% inhibition of OXA-producers) [28]. In our study, only 2% of the isolates were susceptible to sulbactam (in ampicillin/sulbactam). However, this percentage is expected to rise if avibactam or durlobactam were added.

Our study involved a nationwide investigation across various regions of Saudi Arabia. These regions may exhibit distinct patterns of antibiotic usage, leading to differences in susceptibility rates. Although we intended to include more hospitals to expand the sample size, logistical challenges with certain hospitals posed a barrier. Moreover, the prefilled microtiter plates lacked ceftazidime/avibactam, sulbactam/avibactam, sulbactam/durlobactam, and cefiderocol, which are important antibiotics clinically used against MDR *A. baumannii*. Lastly, genotyping could have provided a deeper understanding of the underlying resistance mechanisms, which we recommend for future studies.

## 5. Conclusions

By incorporating samples from seven different hospitals situated across various regions of Saudi Arabia, our study aimed to identify a wide range of *A. baumannii* strains that are prevalent throughout the country, which showed high rates of AMR to antibiotics commonly used in therapy. Data generated from our study are crucial for policymakers and healthcare providers as they offer valuable insights into the current status of AMR in Saudi Arabia. Equipped with this knowledge, policymakers and hospital antimicrobial stewardship committees can make well-informed decisions to develop effective strategies for combating AMR, while clinicians may adapt their treatment plans in accordance with the prevalent susceptibility patterns. The findings from this research will provide a crucial resource for informing public health initiatives, developing antimicrobial stewardship programs, and ultimately improving patient outcomes in the face of rising antibiotic resistance challenges.

## Figures and Tables

**Table 1:** Characteristics of the included hospitals and the number of isolates collected.

Characteristic	Hospital						
	King Abdulaziz University Hospital	King Fahad Hospital of the University	King Khaled Hospital	King Fahad Specialist Hospital	King Fahad Hospital	King Fahad Specialist Hospital	Maternity and Children Hospital of King Salman Medical City
**City**	Jeddah	Dammam	Najran	Qassim	Albaha	Tabuk	Madinah
**Region**	Western	Eastern	Southern	Central	Southern	Northern	Western
**Bed capacity**	879	633	330	600	320	205	500
**Number of ICUs**	5 (99 beds)	5 (77 beds)	4 (56 beds)	4 (49 beds)	4 (64 beds)	2 (43 beds)	3 (117 beds)
**N (%) of isolates**	16 (16)	23 (23)	17 (17)	20 (20)	2 (2)	20 (20)	2 (2)

**Table 2:** Sources of *Acinetobacter baumannii* isolates (*n* = 100).

Source	*n* (%)	XDR *	PDR *
Respiratory	43 (43)	22 (51.2)	19 (44.2)
Skin or soft tissue	16 (16)	9 (56.3)	4 (25)
Urine	15 (15)	10 (66.7)	5 (33.3)
Body fluid	14 (14)	4 (30.8)	8 (61.5)
Blood	8 (8)	3 (37.5)	5 (62.5)
Eye	2 (2)	1 (50)	1 (50)
Cerebrospinal fluid	1 (1)	1 (100)	0 (0)
Ear	1 (1)	1 (100)	0 (0)
***p*-value**	-	0.514/0.400 **	0.425

PDR, pandrug-resistant; XDR, extensive drug-resistant. Note: All collected isolates were reported multidrug-resistant (MDR); therefore, the distribution of MDR isolates is the same as the distribution of the source. * The percentages were calculated based on the total number of isolates from that source. ** After combining isolates of eye, cerebrospinal fluid, and ear into one group due to the very small sample size of each one.

**Table 3:** Antibiotic susceptibility data of *Acinetobacter baumannii*.

Antibiotic	% Susceptibility	MIC_50_	MIC_90_	MIC Range
Amikacin	22	>32	>32	≤4–>32
Ampicillin/sulbactam	2	>64/32	>64/32	≤4/2–>64/32
Cefepime	2	>16	>16	≤2–>16
Ceftazidime	3	>16	>16	4–>16
Ciprofloxacin	0	>2	>2	-
Colistin *	5	>4	>4	1–>4
Doripenem	4	>4	>4	≤0.5–>4
Gentamicin	23	>8	>8	≤1–>8
Imipenem	4	>8	>8	≤1–>8
Levofloxacin	3	>8	>8	≤1–>8
Meropenem	7	>8	>8	≤1–>8
Minocycline	3	>16	>16	≤2–>16
Piperacillin/tazobactam	3	>64/4	>64/4	≤8/4–>64/4
Polymixin B *	1	>4	>4	1–>4
Ticarcillin/clavulanate acid	1	>128/2	>128/2	≤16/2–>128/2
Tigecycline	39	4	>8	≤0.25–>8
Tobramycin	25	>8	>8	≤1–>8
Trimethoprim/sulfamethoxazole	0	>4/76	>4/76	>4/76–>4–76

MIC, minimum inhibitory concentration. * The percentages presented are the percentage of isolates with intermediate resistance since the Clinical and Laboratory Standards Institute M100 document includes intermediate and resistant breakpoints only, which are ≤2 and ≥4 μg/mL, respectively.

## Data Availability

The original contributions presented in this study are included in the article/Supplementary Material. Further inquiries can be directed to the corresponding author.

## Supplementary Materials

The following supporting information can be downloaded at: https://www.mdpi.com/article/10.3390/antibiotics14020209/s1. Table S1: MIC values of all isolates; Table S2: MIC_50_ and MIC_90_ of all antibiotics.

## Abbreviations

The following abbreviations are used in this manuscript:

AMR	Antimicrobial resistance
BMD	Broth microdilution
CLSI	Clinical Laboratory Standards Institute
ICU	Intensive care unit
MDR	Multidrug-resistant
MIC	Minimum inhibitory concentration
XDR	Extensive drug-resistant
PDR	Pandrug-resistant
WHO	World Health Organization
